# Serial electron crystallography for structure determination and phase analysis of nanocrystalline materials

**DOI:** 10.1107/S1600576718009500

**Published:** 2018-08-09

**Authors:** Stef Smeets, Xiaodong Zou, Wei Wan

**Affiliations:** a Department of Materials and Environmental Chemistry, Arrhenius Laboratory, Stockholm University, Stockholm, SE-106, Sweden

**Keywords:** serial crystallography, electron diffraction, structure determination, phase analysis

## Abstract

Serial electron crystallography has been developed as a fully automated method to collect diffraction data on polycrystalline materials using a transmission electron microscope. This enables useful data to be collected on materials that are sensitive to the electron beam and thus difficult to measure using the conventional methods that require long exposure of the same crystal. The data collection strategy combines goniometer translation with electron beam shift, which allows the entire sample stage to be probed. At each position of the goniometer, the locations of the crystals are identified using image recognition techniques. Diffraction data are then collected on each crystal using a quasi-parallel focused beam with a predefined size (usually 300–500 nm). It is shown that with a fast and sensitive Timepix hybrid pixel area detector it is possible to collect diffraction data of up to 3500 crystals per hour. These data can be indexed using a brute-force forward-projection algorithm. Results from several test samples show that 100–200 frames are enough for structure determination using direct methods or dual-space methods. The large number of crystals examined enables quantitative phase analysis and automatic screening of materials for known and unknown phases.

## Abstract 2

Serial crystallography using electron diffraction has been developed as a fully automated method for collecting electron diffraction data from a large number of crystals. It is demonstrated how the data can be used for structure determination and phase analysis.

## 1. Introduction   

With single-crystal electron diffraction (SCED; Kolb *et al.*, 2007 ▸; Zhang, Oleynikov *et al.*, 2010 ▸), it is already possible to collect reasonably accurate and complete diffraction data on nano-sized crystals, and a considerable number of complex crystal structures have now been solved from SCED data directly (Yun *et al.*, 2015 ▸). One of the main limitations of this method is that it is difficult to collect data on materials that are sensitive to radiation damage. Efforts to overcome this problem have focused on reducing the data collection times by continuously rotating the crystal in the electron beam in combination with cooling to increase sample longevity (Nederlof *et al.*, 2013 ▸; Gemmi *et al.*, 2015 ▸; van Genderen *et al.*, 2016 ▸; Wang *et al.*, 2017 ▸). Furthermore, the collection of electron diffraction (ED) data is in many cases not fully automated; a significant amount of time is reserved for finding suitable crystals, and the best crystals are cherry-picked by the operator. The result is that crystal selection is somewhat subjective and it is often unclear how representative the chosen crystal is for a bulk material. Electron diffraction studies are therefore frequently augmented by X-ray powder diffraction (XRPD) data for structure validation and phase analysis, as phase quantification of polycrystalline materials is a problem that remains ubiquitous in electron crystallography.

Serial crystallography is fundamentally different in how data are collected (Standfuss & Spence, 2017 ▸). In a serial crystallography experiment, diffraction data are collected from a large number of randomly oriented crystals and then combined for structure analysis. Such experiments are performed using X-ray radiation at centralized facilities, either X-ray free-electron lasers (Chapman *et al.*, 2011 ▸) or synchrotrons with high-brilliance beamlines (Stellato *et al.*, 2014 ▸). Researchers anticipating the use of electron microscopes for serial crystallography have focused on sample delivery using liquid jets (Deponte *et al.*, 2011 ▸) and calculations on radiation damage (Egerton, 2015 ▸) to study biological samples. Our focus is on the application for materials science (Smeets & Wan, 2017 ▸), where we believe that serial electron diffraction (SerialED) offers new opportunities to study polycrystalline materials. One of the advantages of the serial crystallography approach is that each crystal is exposed only once to collect a diffraction pattern before any significant radiation damage can occur. This makes it ideal for the collection of data on radiation-sensitive materials and overcomes some of the limitations associated with the SCED data collection methods that require extended exposure of the same crystal. The other advantage of SerialED is that it enables data to be gathered from a large number of crystals automatically, which can give some bulk information about the material for phase analyses and allow computerized screening for crystals.

There are several properties of transmission electron microscopes that make them well suited for serial crystallography: (1) electrons diffract much more strongly than X-rays and high-quality electron diffraction patterns can be obtained from crystals of a few tens of nanometres in size; (2) crystals can be observed directly in imaging mode, eliminating the randomness typically associated with serial crystallography experiments, so that useful information can be extracted from nearly every frame; and (3) there is an electron microscope available in many laboratories. Modern electron microscopes are computer controlled, such that the entire data collection process can be fully automated through software. Ever since computer-controlled microscopes were introduced, researchers have made use of the software interfaces to automate experiments. Early efforts focused mostly on autotuning to minimize the electron dose (Koster *et al.*, 1989 ▸) for tomography studies on biological samples (Dierksen *et al.*, 1992 ▸). Later, interfaces started to emerge in Python (Kisseberth *et al.*, 1997 ▸) and Java (Hadida-Hassan *et al.*, 1999 ▸) for remote control of electron microscopes. The former eventually evolved into the Leginon system for automated mol­ecular microscopy (Suloway *et al.*, 2005 ▸, 2009 ▸), which allows data to be collected on single particles and is highly relevant today in the cryo-electron microscopy community (Bai *et al.*, 2015 ▸). Examples related to electron diffraction include automated diffraction tomography (Kolb *et al.*, 2007 ▸), which automatically tracks the position of the crystal *via* software during data collection, the software control of the electron beam to enable precession (which typically requires expensive hardware) using a digital sampling method (Zhang, Grüner *et al.*, 2010 ▸), rotation electron diffraction (Zhang, Oleynikov *et al.*, 2010 ▸; Wan *et al.*, 2013 ▸), which combines a computer-controlled goniometer and beam tilt for fine sampling of the reciprocal space, and automated orientation and phase mapping using scanning diffraction techniques (Rauch *et al.*, 2010 ▸).

Here, we present a method to collect SerialED data on a transmission electron microscope. To make this possible, we have developed a software library that controls both the transmission electron microscope and the camera to fully automate the data collection, and we discuss the instrumentation and data acquisition procedures that make this possible. We have also developed algorithms to process and merge these data, and show that the method can be applied for structure determination and phase analysis of nano-sized crystals (dimensions <1 µm).

## 2. Serial electron crystallography   

**Figure 1:**
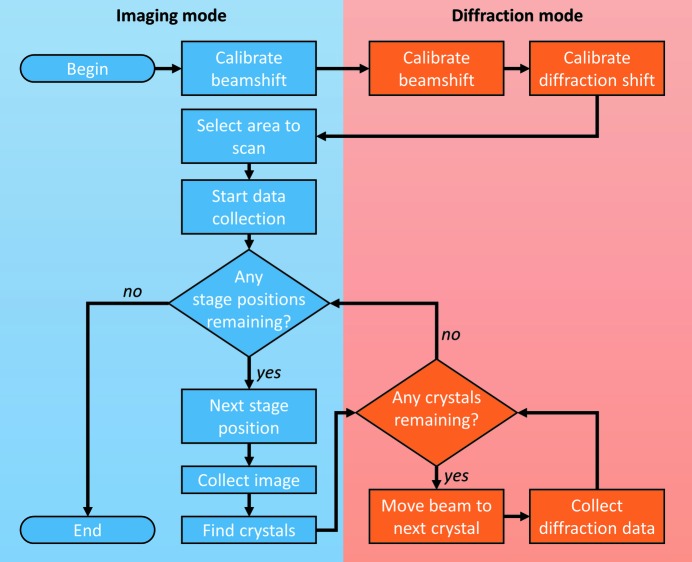
Flowchart illustrating the SerialED data collection procedure. The blue and red colours indicate the operations that are performed in imaging and diffraction modes, respectively.

**Figure 2:**
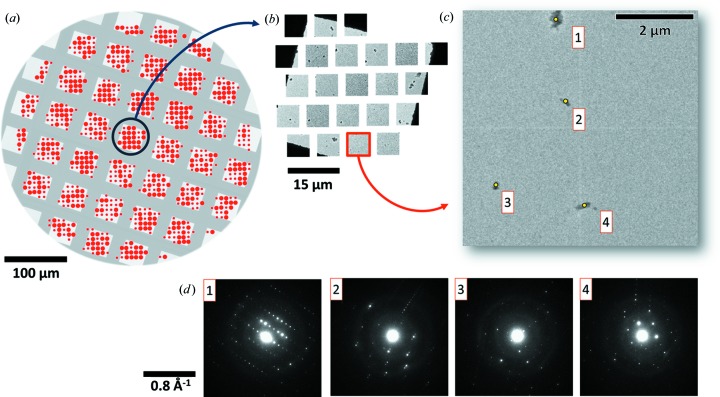
(*a*) Low-magnification overview of the area on the sample grid where ED data are collected. Each spot corresponds to a position of the sample stage, and an image is taken at each position. The larger red spots indicate images in which crystals have been detected. (*b*) Enlarged view of some of the images. (*c*) Example image where four crystals have been detected, and (*d*) their corresponding diffraction patterns.

Most modern transmission electron microscopes allow for (near) full control over the goniometer (sample stage), lenses and camera. We make use of the fact that it is possible to switch between imaging and diffraction modes on the fly, and that the electron beam can be translated or tilted, focused or spread *via* software. Goniometer movement is slow and imprecise compared to electron beam shift, which is fast and reliable. Therefore, our strategy to collect SerialED data combines goniometer translation with electron beam shift, which allows the entire sample stage to be probed. To make data collection efficient, crystals are identified in imaging mode using a low magnification with a parallel beam illuminating a large area on the grid. Once crystals have been identified, the microscope is switched to diffraction mode, and the electron beam is focused on each crystal so that ED data can be collected. Fig. 1 ▸ shows a flowchart of the general data collection procedure, and Fig. 2 ▸ shows an example of what data collection looks like. The procedure can be subdivided into three major steps: (1) setup and calibration, (2) crystal detection in imaging mode, and (3) collecting ED data in diffraction mode. The calibration of the electron beam is required to ensure that crystals can be hit by the focused electron beam and that the diffraction patterns are centred on the detector. After crystals have been located and identified in image mode at low magnification, they are probed in diffraction mode using a beam with a pre-defined size (parallel or with a small convergence angle). Each of these steps is detailed below, and the implementation of the method on our JEOL JEM-2100-LaB6 microscope is described. As the elements of the microscope required by the method are the beam deflectors and sample stages which are available on all modern microscopes, we expect the method to work on microscopes from other manufactures with minimal adaptation.

### 2.1. Microscope calibration   

The data collection routine described here uses the beam shift deflectors (first condenser lens deflector, CLA1) and diffraction shift (projector lens deflector, PLA) to control the position of the beam, so that a focused quasi-parallel beam can be used to probe individual crystals reliably and that the resulting diffraction pattern is centred on the detector. This is achieved by defining three transformations (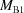, 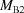 and 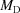). Here, *M* is a 2 × 2 matrix that defines the affine transformation as a combination of scaling and rotation:
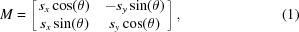
where 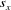 and 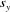 represent the scaling parameters along the *x* and *y* directions, and θ is the rotation angle around the normal to the plane defined by *x* and *y*.

The first matrix, 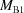, defines the position of the incident beam on the detector in imaging mode as a function of the beam shift deflectors. We define the difference in the value of the beam shift deflectors 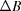 in relation to the difference in pixel coordinates 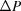, with respect to pre-defined reference positions for both:
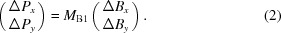
The calibration is performed by focusing the beam to the required size (usually 100–1000 nm on our setup) and centring the beam on the detector. We have automated the calibration procedure by programming the microscope to collect 25 images from a five by five grid of positions for the incident beam. The pixel shifts are determined from cross-correlation maps with the image at the reference position (Fig. S1*a* of the supporting information), and the values of 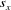, 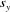 and θ can be found *via* a least-squares fit.

The other two transformations, 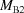 and 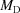, define the position of the primary beam in diffraction mode as a function of the beam shift and projector lens deflectors, respectively, and ensure the positional stability of the incident beam. A beam shift applied to the incident beam is often accompanied by a beam tilt which influences the position of the primary beam in the diffraction pattern, often moving it out to the side or even out of the view of the detector. The calibration to correct for this is performed in diffraction mode and carried out in two steps. First, the effect of the beam shift deflectors on the position of the primary beam is measured. Second, the effect of the diffraction shift deflectors on the position of the primary beam is measured, so that the movement from the beam shift deflectors can be compensated. The differences in the values of the deflectors, and the corresponding position observed as a shift of the pixel coordinates 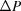 of the primary beam in the diffraction pattern, can be described as
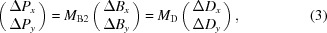
where 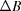 and 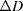 are the offsets of the beam shift (CLA1) and diffraction shift (PLA) deflectors corresponding to their pre-defined neutral positions, and 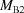 and 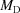 are the corresponding affine transformation matrices. The pixel shifts 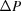 are determined from cross-correlation maps with the image at the centred position of the primary beam. Then, the value of the diffraction shift deflectors required (defined as an offset) to compensate the effect of moving the incident beam using the beam shift deflectors can be calculated as
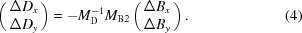
The values of 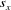, 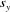 and θ that define 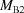 and 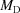 are found in the same way as for the beam shift in imaging mode (Figs. S2 and S3).

Note that it is possible to achieve pure beam shift by combining beam shift with a beam tilt deflector. However, this adds complexity to the routine and makes a negligible difference to the data as all patterns are from crystals in random orientations, and the tilt and shift contributions can be isolated to some extent by aligning the tilt and shift compensators. Another point is that on our microscope there is some hysteresis that arises when manual and programmatical adjustments to the lenses are mixed, which affects the observed position of the incident beam on the detector. In order to eliminate the hysteresis, we simply toggle between diffraction and imaging mode several times before starting the calibration to relax the lenses and reset the observed position of the incident beam to its neutral position. On our microscope this noticeably enhances the stability of the incident beam during data collection. A good calibration and alignment of the microscope is necessary to perform the experiments. The calibration depends on the daily alignment of the microscope and, in particular, the value of the condensor lens (CL3) and the eucentric height. For this reason, the calibration is performed routinely before every experiment. A discussion on the stability of the calibration from over 65 experiments can be found in the supporting information (§S1).

### 2.2. Crystal detection in imaging mode   

In imaging mode, a survey of the sample grid is made in the first step. The data collection procedure is initialized by selecting the area of the sample stage to start, and by defining a scan radius (usually 100–200 µm). The defined area is subdivided into a grid of equally spaced positions of the sample stage. Fig. 2 ▸(*a*) shows an overview of what a map of the data collection may look like. The sample stage is moved to each grid position and an image is taken (Fig. 2 ▸ *b* and 2 ▸ *c*). Images of the Cu grid are detected by comparing the mean value of the intensities with a preset threshold value. If the mean is lower than the threshold, the image is rejected, and the sample stage is moved to the next position.

**Figure 3:**
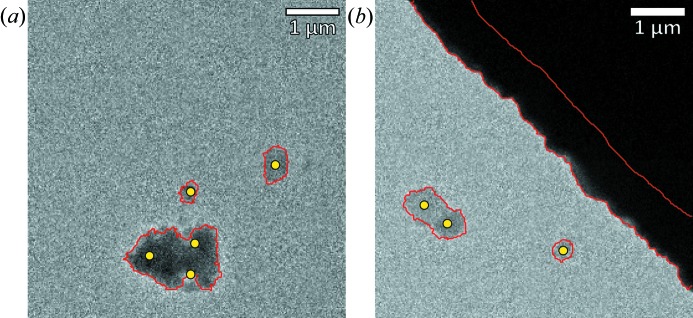
Examples of the results obtained from the crystal-finding algorithm. Red lines indicate the contours of the connected areas as found by the image-segmentation algorithm. Yellow dots indicate the *xy* coordinates used to position the incident beam for data collection. In (*b*), an area containing the Cu grid is shown. Although it is detected by the segmentation algorithm, it is recognized as a Cu grid and therefore ignored for further processing.

To detect if there are any crystals present in the image (*e.g.* Fig. 2 ▸ *c*), segmentation is performed by applying a local threshold filter followed by a series of erosion and dilation operations to clean up the image and remove small objects. An unforeseen benefit of using local thresholds for image segmentation is that it favours the edges of large clusters of crystals. This avoids diffraction patterns being taken in the centre of these clusters, which are typically too thick for the electron beam to penetrate. Crystals then are detected by looking for regions of connected pixels with nonzero intensity values. Fig. 3 ▸ shows in red the contours of what these regions may look like. If a connected region touches the image edge and has a very sharp intensity histogram, it is recognized as a copper edge and rejected as a crystal [*e.g.* the dark corner in Fig. 3 ▸(*b*)]. Otherwise, the area (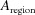) of each connected region is calculated. If the area is small enough (below a predefined threshold value, *e.g.* 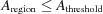), the connected region is assumed to be a single crystallite, and its centroid coordinates are added to the list of points to be probed. If it is larger, the connected region is assumed to be a collection of crystals, and *k*-means clustering is used to find *n* equally spread coordinates, where 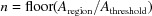. The yellow dots in Fig. 3 ▸ show the extracted crystal coordinates and correspond to either the centre of the crystal area or a series of points as obtained from the *k*-means clustering. If no crystals are detected (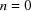), the stage is moved to the next position. If crystals are detected (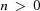), the routine switches to diffraction mode for data collection.

### 2.3. Diffraction mode   

In the next step, diffraction data are collected on each of the crystals (Figs. 2 ▸ *c* and 2 ▸ *d*). This is done by switching the microscope to diffraction mode and focusing the beam by changing the brightness control (third condensor lens, CL3) to the predefined value to achieve the desired size of the beam. Because the position of the beam has been calibrated, the pixel coordinates of the crystals can be mapped to the corresponding values of the beam shift deflectors to centre the beam on each crystal, and the corresponding values for the diffraction shift deflectors are applied to ensure the primary beam is close to its centred position. After diffraction data have been collected on all crystals found in the current image, the routine switches the microscope back to image mode, the sample stage is moved to the next position and the procedure is repeated until all target stage positions have been exhausted.

### 2.4. Implementation   

Our experiments were performed on a JEOL JEM-2100-LaB6 at 200 kV equipped with a 512 × 512 Timepix hybrid pixel detector (55 × 55 µm pixel size, QTPX-262k, Amster­dam Scientific Instruments). The use of a small condenser lens aperture (50 or 70 µm) in combination with a small spot size (large spot number, 4 or 5 on our microscope) serves to reduce the intensity of the incident beam, to minimize the convergence angle (making the beam as parallel as possible) and to isolate the central part of the beam, which comes from the tip of the filament. We observed that each of these properties helped to optimize the calibration and stability of the primary beam to minimize frame-to-frame variations. Images were taken in image mode (MAG1) at a magnification of 2500×. This is the lowest magnification available on our microscope without going into the ‘low mag’ mode where the objective lens is switched off. This magnification encompasses an area of 6.0 × 6.0 µm and is used to include as many crystals as possible. The convergence angle of the incident beam can be adjusted *via* the brightness control (condenser lens, CL3). In this way, the size of the incident beam can be controlled precisely. In our experiments, we tuned it so that an area with a diameter in the range of 200–500 nm was illuminated on the sample. For data collection, a standard calibration routine is performed first to obtain a mapping of the pixel positions to specific values of the deflectors (as described above). The diffraction pattern is focused using the diffraction focus lens (IL1). The camera length is usually set at 300 mm, because this gives a maximum resolution (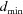) of approximately 1.0 Å on our detector, which is suitable for structure determination. For the images, we used an exposure time of 0.3–0.5 s, which gives an image of sufficient contrast for crystal identification. For the diffraction patterns an exposure time of 0.1–0.2 s was used.

To carry out the data collection procedure, we built a custom data collection software that controls both the microscope and the camera (Smeets *et al.*, 2017 ▸), implemented in Python 3.6. For microscope control, we implemented a wrapper around the application programming interface (API) for control of the JEOL microscope, which was inspired in part by the PyScope library (Suloway *et al.*, 2005 ▸). We implemented interfaces to the Timepix and Gatan OriusCCD cameras using their respective APIs provided by the manufactures. These interfaces were abstracted away in generic control interface objects so that other microscopes and cameras may be included in the future. Data processing was performed using the numpy (http://www.numpy.org/), scipy (http://www.scipy.org/), scikit-image (Walt *et al.*, 2014 ▸), lmfit (Newville *et al.*, 2014 ▸), pyXem (http://github.com/pyxem/pyxem) and HyperSpy (de la Peña *et al.*, 2017 ▸) libraries.

## 3. Data processing   

### 3.1. Image processing   

Our Timepix detector is assembled from four modules with a resolution of 256 × 256 each, where the pixels connecting the modules are three times longer or wider (165 µm instead of 55 µm). To ensure the correct geometry for further processing, the images are converted to a 516 × 516 array (see also Nederlof *et al.*, 2013 ▸). The intensity of these larger pixels is corrected for by the flatfield correction, but they may also be masked at a later stage. A flatfield correction is applied to correct for the slight variation in pixel response. Lens distortions (Capitani *et al.*, 2006 ▸) are corrected by applying an affine transformation to the image. On our microscope, we observe an elliptical distortion with an eccentricity of 0.22.

### 3.2. Finding the position of the primary beam   

The position of the primary beam (corresponding to the centre of the diffraction pattern) is found by applying a Gaussian filter to the entire image with a large enough standard deviation (usually 10–30). The position of the primary beam will then be at the pixel with the largest intensity value.

### 3.3. Background subtraction and peak identification   

Background subtraction for peak identification is performed using a median filter, as described by Barty *et al.* (2014 ▸). For the samples in this study, the window for the filter was chosen to be 19 pixels wide, which defines a box of approximately three times the number of pixels for a typical peak.

Peak detection is then performed by calculating the difference between two Gaussian convolutions of the input image with standard deviations of, respectively, 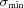 and 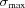, where the value of 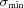 is typically between 2 and 5 and 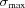 between 3 and 8 (ensuring that 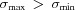). Reflections are identified by searching for regions of connected pixels that have an intensity value larger than a threshold value *T* (usually 1–3). The intensities of the pixels belonging to the background or any regions consisting of less than 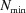 pixels (usually 10–30) are set to zero in order to remove noise for the orientation-finding algorithm.

### 3.4. Orientation finding and intensity extraction   

Orientation finding is performed using a brute-force forward-projection algorithm based on the one described by Rius *et al.* (2015 ▸). This algorithm was chosen because it is fast and its implementation straightforward. It requires the lattice parameters as prior information, and it is helpful if the Laue class or space group is also known. Both can be obtained from a SCED data collection (Kolb *et al.*, 2007 ▸; Zhang, Oleynikov *et al.*, 2010 ▸) on an isolated crystal (if not already known). Alternatively, algorithms for obtaining the unit cell from randomly oriented crystals directly also exist (Jiang *et al.*, 2009 ▸). Then, a library of reflection projections is generated for all unique crystal orientations for the given unit cell and space group (spaced roughly 0.03 radians or 1.7° apart to achieve sufficient coverage of the reciprocal space). For each orientation, the pixel positions on the camera of the reflections in Bragg condition are calculated, and a summation of the intensities at all of these pixel positions is made (the ‘score’ value). Before the summation, the intensities are multiplied by the excitation error (*i.e.* the distance of the reflection to the Ewald sphere). The goal of the algorithm is to find the orientations with the largest score.

**Figure 4:**
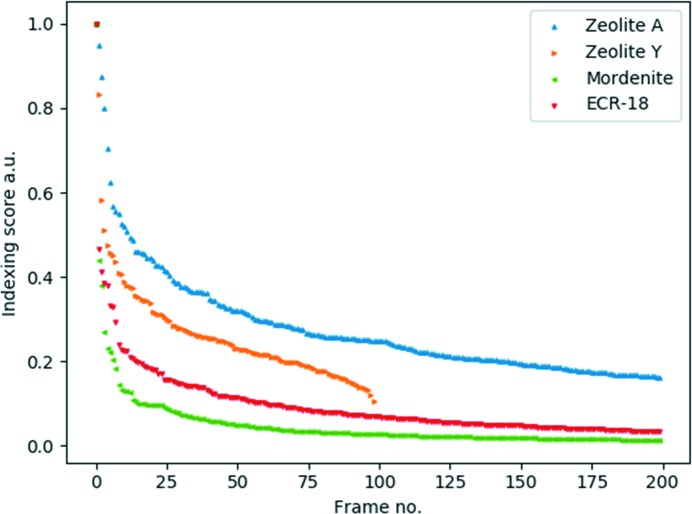
Distribution of the indexing scores obtained from the orientation-mapping routine, showing the best 200 scores (corresponding to the 200 ‘best’ frames) for zeolite A, mordenite and ECR-18, and the best 100 scores for zeolite Y. For comparison, the values were divided by the largest value in each data set.

To account for the fact that the algorithm tends to favour projections with a very dense distribution of reflections (*i.e.* low-order zone-axis patterns), the score is updated by multiplying it with the ratio of nonzero intensities to zero intensities (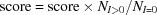). The solutions are ranked by their score, and the orientations, beam centre and/or scale of the highest-ranked solutions (typically 25) are optimized using a least-squares minimization with the inverse of the indexing score (1/score) as the objective function. The orientation with the highest score is assumed to represent the best solution. Fig. 4 ▸ shows the distribution of the highest scores obtained from the 200 best frames (as judged by the indexing score). Intensity extraction is performed by simply taking the largest pixel intensity within a radius of three pixels from the predicted reflection position of the optimized orientation.

### 3.5. Merging   

Merging electron diffraction data from a large number of snapshots is difficult, because of variations in the diffracting volumes, crystal quality, Debye–Waller factors, flux of the incident beam and reflection partiality. We have recently developed an algorithm named SerialMerge that can overcome some of these problems (Smeets & Wan, 2017 ▸). Instead of modelling the diffraction processes that relate the structure factors to the intensities of the spots observed in the diffraction pattern, the SerialMerge method retrieves the most likely reflection ranking. As a result, it is tolerant to errors in the diffraction intensities, which are inevitable in electron diffraction because of dynamical scattering, and the issue of scaling is avoided altogether. The downside is that the algorithm produces the most likely ranking of the reflections only; the values of the reflection intensities are lost in the merging procedure (because it optimizes for rank, not intensity). To recover the intensities, we apply a histogram-matching routine, where the extracted intensities or calculated intensities from a related material are used. These intensities are then ranked in a histogram and matched to the obtained ranking. We find that using a histogram of calculated intensities from a related material produces better results (see §4).

Our initial tests using SerialED data showed that structure determination failed if all reflections are included for merging, because not all reflection positions predicted by the orientation-finding algorithm were actually observed, resulting in many reflections erroneously being assigned an intensity value of zero. Therefore, all extracted reflections with an intensity of zero are ignored for merging.

## 4. Application to test samples   

**Figure 5:**
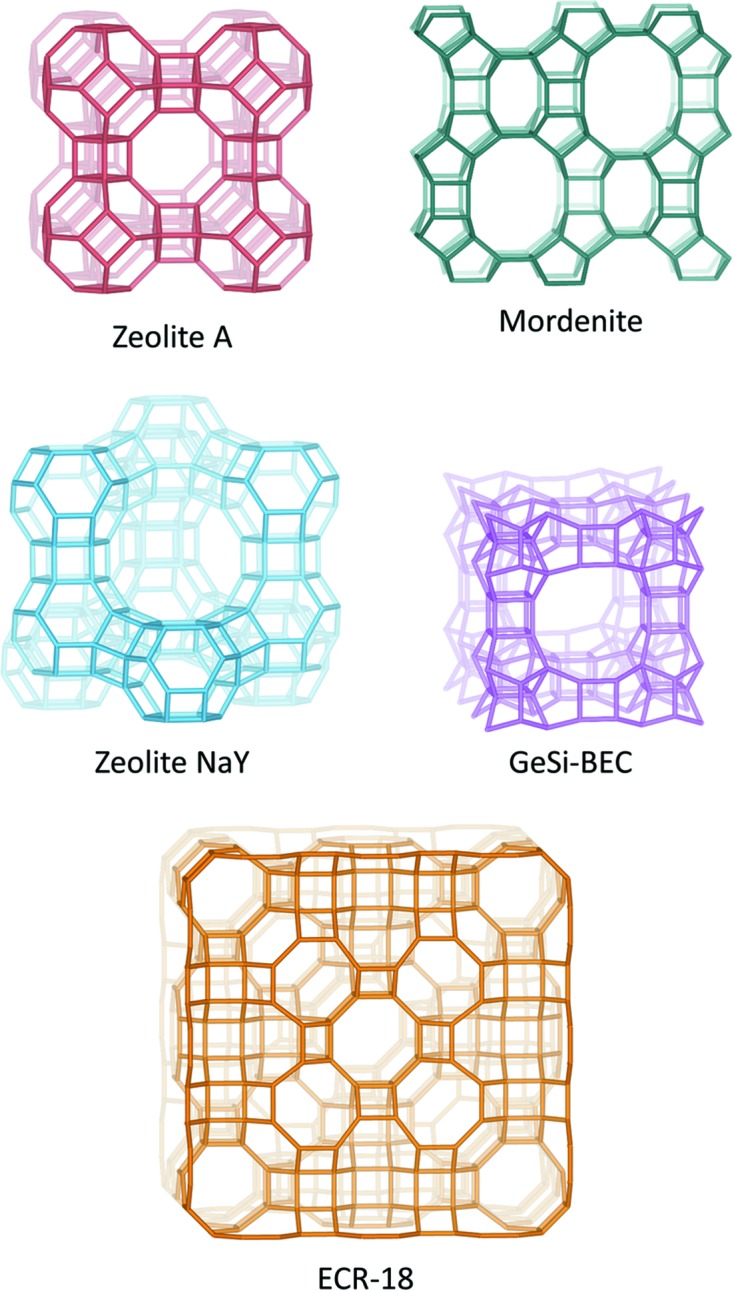
Structures determined using the SerialED method. The structures of zeolites A and Y could be determined using direct methods implemented in *SHELXS*, and mordenite, GeSi-**BEC** and ECR-18 were determined using the dual-space method implemented in *FOCUS*. O atoms have been omitted for clarity.

For testing purposes, SerialED data were collected on zeolites A (**LTA**), Y (**FAU**), GeSi-**BEC** (Corma *et al.*, 2001 ▸), mordenite (**MOR**) and ECR-18 (**PAU**; Vaughan & Strohmaier, 1999 ▸), as well as a coordination polymer Co-CAU-36 (Wang *et al.*, 2018 ▸). The structures of the zeolites are well known and were determined previously by single-crystal or powder diffraction. Each of them is assigned a three-letter code by the Structure Commission of the International Zeolite Association to represent the framework type of the zeolite (Baerlocher *et al.*, 2007 ▸). The corresponding framework structures are shown in Fig. 5 ▸. A summary of the crystallographic details and structure determination by SerialED is given in Table 1 ▸, and of the data collection statistics in Table 2 ▸. GeSi-**BEC** is discussed in the supporting information (§S2).

### 4.1. Sample preparation   

Zeolite samples were prepared on Cu grids with continuous carbon film (CF400-Cu-UL from Electron Microscopy Sciences). Although grids with continuous carbon film will give rise to increased background signal, the benefit is that there are only two levels of contrast (carbon film, crystals) in the images, as opposed to three levels (vacuum, carbon film, crystals) with conventional lacey carbon. This makes it significantly simpler to perform image segregation to identify the position of the crystals on the grid. Samples were prepared by crushing powder in a mortar. The samples were suspended in ethanol and sonicated for 2–8 min. It is preferred to have a dense distribution of crystals on the grid, so we tended to make a relatively concentrated suspension. One to three droplets were then added to the grid; excess liquid was removed with a paper tissue, after which the ethanol was allowed to evaporate.

### 4.2. Structure determination   

#### 4.2.1. Zeolite A   

**Figure 6:**
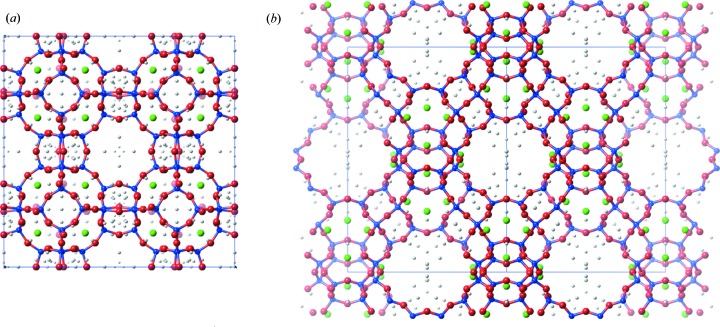
Structure obtained from *SHELXS* after the *Q* peaks have been assigned for (*a*) zeolite A and (*b*) zeolite Y. Si is coloured blue, O red and Na green. The remaining *Q* peaks are shown in grey.

Zeolite A is a small-pore zeolite that was first discovered in 1956 (Reed & Breck, 1956 ▸) and is widely used as an ion-exchange agent. Its framework structure (SiO_2_) consists of three independent atoms and several cations (*e.g.* Na^+^, K^+^) that are occluded in its three-dimensional channel system. It has a well defined cubic crystal structure (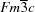; *a* = 24.610 Å) and forms as roughly 1 µm-sized crystals. It therefore makes an excellent sample for testing the method. Several series of data have been collected on this sample. In one of the experiments, 442 images were collected by scanning an area of 300 × 300 µm. The number of particles detected in these images was 1107, and diffraction data were recorded on each of them. The patterns were processed as described above and run through the orientation-finding algorithm. Reflections were ranked and merged *via* the SerialMerge algorithm, using the intensities observed in the 200 frames with the highest indexing scores. In this case, we applied a histogram of the observed intensities to the ranked reflections. The structure was solved straightforwardly from the resulting data set using direct methods implemented in *SHELXS* (Sheldrick, 2008 ▸). The Si atom and its four tetragonally bonded O atoms were located by *SHELXS* directly (albeit mislabelled), and two adsorbed Na^+^ ions were among the strongest *Q* peaks (Fig. 6 ▸ *a*). There are several more observed *Q* peaks. Although these may correspond to other adsorbed species in the channel system, we were hesitant to overinterpret these data. Table 3 ▸ shows the shifts of the atomic positions with respect to the reference crystal structure obtained from single-crystal X-ray diffraction data (Grämlich & Meier, 1971 ▸). The deviations are 0.13 (6) Å on average for the framework atoms (Si, Al, O) and 0.35 Å for the Na^+^ ion in the pore, showing that the obtained solution is quite close to the expected one. No attempts were made to refine the crystal structure against the SerialED data. However, the obtained crystal structure is close enough to the reference structure to provide a good starting point for structure refinement from, for example, XRPD data.

#### 4.2.2. Zeolite Y   

Zeolite Y (**FAU**) is an aluminosilicate zeolite that was discovered in 1964 (Breck, 1964 ▸) and forms as 1 µm-sized crystals with a cubic crystal structure (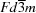; *a* = 24.740 Å). It has important industrial applications in fluid catalytic cracking, in part because it is cheap and flexible to produce, thermally stable, and one of the lowest-density zeolites owing to its large accessible cages. The latter may also make its structure determination challenging. The largest data set we collected covered an area of 400 × 400 µm and consists of 2506 diffraction patterns. The hkl files from the 100 frames with the highest indexing scores from the orientation-finding algorithm were merged *via* the SerialMerge algorithm, using the histogram of observed reflections as a source of reflection intensities. The structure of zeolite Y could then be solved using direct methods (*SHELXS*), and all five atoms (one Si and four O atoms) could be located from the list of *Q* peaks directly. Additionally, two more *Q* peaks could be assigned to Na^+^ ions. To evaluate the quality of the solution, we looked at the bond distances. Here we find that the *T*—O distances range from 1.55 to 1.73 Å, in line with what is expected for Si—O (1.61 Å) and Al—O (1.73 Å). The *T*—O—*T* angles of 133–140° and O—*T*—O angles of 104–115° are in line with the expected values (∼145 and ∼109.5°, respectively). This accuracy is reflected in the positions of the atomic parameters when compared with the published coordinates of zeolite Y (Hriljac *et al.*, 1993 ▸), which deviate by 0.14 (4) Å on average for the framework atoms and 0.29 (8) Å for the Na^+^ ions in the pore (Table 3 ▸). The published coordinates contain a third Na^+^ ion with a low occupancy on a special position (16*c*), which we did not locate among the *Q* peaks.

#### 4.2.3. Mordenite   

**Figure 7:**
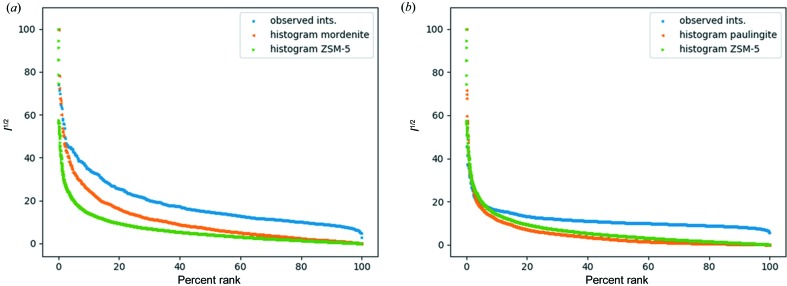
Histogram normalization for (*a*) mordenite and (*b*) ECR-18, showing the histogram of the observed intensities in blue, that of ZSM-5 which was used for the histogram matching in green and the ideal histogram corresponding to the framework structure in orange.

**Figure 8:**
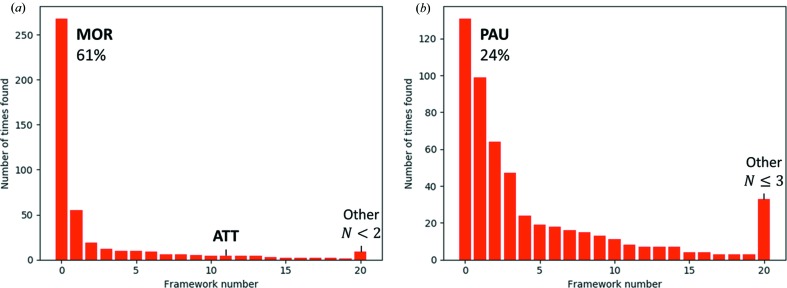
Histograms showing the distribution of the 20 most frequently found frameworks for (*a*) mordenite (**MOR**) and (*b*) ECR-18 (**PAU**).

Synthetic mordenite is a large-pore zeolite that is widely used in the petrochemical industry for acid-catalysed isomerization of alkanes and aromatics. It has an orthorhombic (*Cmcm*) crystal structure and presents significant challenges in data collection. We discuss here a data set consisting of 694 diffraction patterns collected over an area of 200 × 200 µm. The lower symmetry (compared with the cubic samples above) has profound consequences on the orientation-finding algorithm. First of all, preferred orientation is a factor that should be considered, because it often leads to a missing cone in the data. The missing wedge is a common problem in SCED, because it limits the completeness of the data that may be collected. It is well known that the completeness of the data set is among the most important parameters for structure determination (Klein, 2013 ▸). Second, the number of unique orientations required to index an orthorhombic crystal is about three times higher than for the cubic case. Although this is not a problem by itself, it highlights one of the issues with the orientation-finding algorithm: a solution to the orientation problem is always found. False positives artificially increase the completeness but will actually reduce the data quality of the merged data set. Using the best 62 frames on the basis of the orientation-finding score and the observed reflections for the histogram matching, we were able to obtain a data set with an estimated completeness of around 60%. As a result of the low completeness, *SHELXS*/*SHELXT* (Sheldrick, 2008 ▸, 2015 ▸) produced partial (but mostly incorrect) models only. Using the zeolite-specific program *FOCUS* (Smeets *et al.*, 2013 ▸), which relies on *a priori* information about zeolites and includes a built-in framework search, we were still unable to determine the framework structure from the data directly. We suspected the distribution of intensities may be a problem and therefore matched the intensity histogram with that of archetypical zeolite ZSM-5 (**MFI**) (Fig. 7 ▸ *a*). This significantly improved the data quality, and the correct structure was immediately found by *FOCUS* using default parameters based on the rules outlined by Smeets *et al.* (2013 ▸). The histogram of solutions (Fig. 8 ▸ *a*) produced over 10 000 cycles shows that the data are rather selective towards the right framework structure, which was produced in the majority (61%) of the successful trials. Despite the better data quality, *SHELXS*/*SHELXT* did not give the structure solution from the new data.

#### 4.2.4. ECR-18   

ECR-18 is the synthetic analogue to the naturally occurring zeolite paulingite (Vaughan & Strohmaier, 1999 ▸). It crystallizes in the cubic crystal system (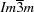) and has the largest unit-cell parameters that we have tested (*a* = 35.08 Å, *V* = 43169 Å^3^). We collected several samples, the best one of which was collected over an area of 300 × 300 µm, resulting in 780 diffraction patterns. The orientation-finding algorithm here is impaired by the large unit-cell parameters and the relatively low resolution of the camera (512 × 512 pixels). The half-width of the reflections is around 5–10 pixels, which is comparable to the separation of the reflections. It would be beneficial to reduce the camera length, so that the reflections can be modelled better. However, on our camera (with 512 × 512 pixel dimensions), doing so would limit the maximum *d* spacing of the data that can be collected. The best 83 diffraction patterns were merged, giving a data set that is 87% complete up to a resolution of 1.35 Å. Structure factors calculated for ZSM-5 were used to provide intensities for the histogram-matching routine (Fig. 7 ▸ *b*). These data were not good enough for structure determination using direct methods. The structure of ECR-18 could be solved using *FOCUS* for 567 072 trials over the course of two days to produce 539 framework structures, and the most frequently occurring solution corresponds to the expected **PAU** framework type (Fig. 8 ▸ *b*).

### 4.3. Phase analysis   

Phase analysis of polycrystalline materials is of high interest for industrial applications, for example screening and quality control. Phase analysis is typically the domain of XRPD. However, several characteristics that are inherent to the material can make the analysis of XRPD data problematic, such as preferred orientation, reflection overlap from materials with low crystal symmetry and/or large unit cells, and the fact that some materials contain multiple phases. In this section, we show how SerialED can potentially be used for phase analysis of polycrystalline materials.

**Figure 9:**
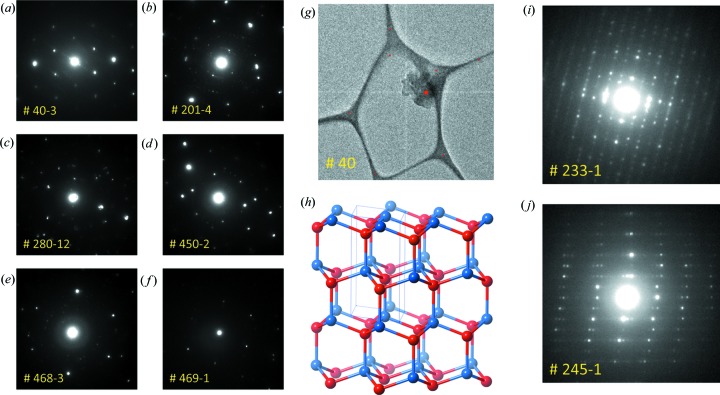
(*a*)–(*f*) Diffraction patterns for the six impurity patterns found in a powder sample of Co-CAU-36. (*g*) Image of the crystal that was used to collect SCED data with the continuous rotation method, corresponding to (*a*). (*h*) Wurtzite structure of the CoO impurity. (*i*), (*j*) Selected diffraction patterns representative of the Co-CAU-36 crystals.

Co-CAU-36 is a Co-based metal–organic framework (MOF) (Wang *et al.*, 2018 ▸) whose structure was first determined using SCED data collected with the method described by Wang *et al.* (2017 ▸). As part of a series of tests on various materials, we collected SerialED data on Co-CAU-36. Much to our surprise, we noticed several diffraction patterns that were obviously different (Figs. 9 ▸ *a*–9 ▸ *f*) from the typical MOF crystals (*e.g.* Figs. 9 ▸ *i* and 9 ▸ *j*). Out of the 1202 images we collected, only about 500 contain diffraction patterns. The reason for this is that we used a lacey carbon grid for the data collection, which resulted in a large number of diffraction patterns being taken of the carbon film. Out of these 500 diffraction patterns, six patterns with significantly different diffraction patterns could be identified by visual inspection (Figs. 9 ▸ *a*–9 ▸ *f*). The positions of the crystals are stored when diffraction data are collected, so it was possible to go back to the crystals in order to further investigate them. Two of the crystals were sufficiently isolated (the other four were too close to the Cu grid or other crystals) and were used to collect SCED data using the continuous rotation method as implemented in the program *Instamatic* (Smeets *et al.*, 2017 ▸). For one of them (Fig. 9 ▸ *g*), we were able to collect a rotation series from 34.45 to −13.79° with an oscillation angle of 0.23° and exposure time of 0.5 s per frame. The data could be indexed using the program *XDS* (Kabsch, 2010 ▸), giving a hexagonal unit cell (*a* = 3.10, *c* = 5.45 Å; Table 4 ▸). Although the data were not good enough for structure determination using direct methods, a quick search of the Inorganic Crystal Structure Database (ICSD; Belsky *et al.*, 2002 ▸) revealed a match of the unit cell to a wurtzite-like structure (cobalt oxide, space group 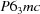; Fig. 9 ▸ *h*). The chemical composition of this crystal was confirmed to be cobalt oxide using a quick EDS measurement. On the basis of these data, we can therefore cautiously estimate the powder sample to consist of 99% Co-CAU-36, containing a trace impurity (<1%) of cobalt oxide. In this case, the identification was possible because the impurity is visually very different from the main phase. This indicates that phase separation of trace impurities is in principle possible, but may require further development for automation.

## 5. Discussion   

One of the key problems to overcome is finding the crystal orientations reliably, so that the diffraction patterns can be indexed. Unfortunately, there are no mature algorithms or programs that unequivocally find the correct orientation for a single electron diffraction snapshot from a randomly oriented crystal. The cut of the Ewald sphere through the reciprocal lattice is essentially flat. This eliminates the use of software that has been effective for indexing large quantities of X-ray snapshot data so that forward-modelling and brute-force routines are often the only options (*i.e.* Rauch & Dupuy, 2005 ▸). This means that the lattice parameters (and space group) should be known beforehand or found independently (*e.g.* from an XRPD pattern or SCED data set).

Fig. 4 ▸ shows the distribution of indexing scores for the data used for structure determination. There are typically a few frames with very high scores, often corresponding to nearly perfectly aligned zone-axis patterns, and a long tail corresponding to frames with no diffraction data, resulting in very low scores. These scores indicated that crystals of all sorts of qualities are present in the data set. Usually, for electron microscopy studies, the very best crystals are chosen for analysis, and these may not be representative for the bulk material. The advantage of SerialED is that it is less biased. At some point, however, a line should be drawn between a ‘good‘ and ‘bad‘ diffraction pattern. Crystals can be too thick, overlapping with each other or amorphous or may simply diffract poorly. We use the orientation-finding algorithm as an indicator of quality, based on how well the diffraction patterns are indexed. Data are selected by setting a minimum threshold for the indexing score or taking the top *N* solutions, because accurate and, perhaps more importantly, reliable orientations are needed for structure determination. The SerialMerge algorithm (Smeets & Wan, 2017 ▸) was developed with this in mind and is insensitive, to a certain extent, to incorrectly indexed reflections or diffraction patterns. However, too many wrongly indexed patterns will be detrimental to structure determination.

There are other subtle variations between diffraction patterns that exacerbate the problem of orientation finding. One of them is that a convergent beam is used for data collection. As the scale of the diffraction patterns is more sensitive to the sample height compared to the case of a parallel beam, fluctuations in the scaling of the pattern (as expressed by the pixel size in px Å^−1^, related to camera length) are difficult to avoid. This problem can be alleviated using the nanometre-sized parallel beam that is available on electron microscopes with field-emission guns.

Another problem is sample preparation. The zeolites we have studied tend to form clusters that are undesirable. The ideal sample would consist of particles roughly 100–500 nm in size, which are distributed densely and evenly on the grid, without overlap or preferred orientation. Crystals with preferred orientations will adversely affect the completeness of the data that can be collected. This is a common problem in electron microscopy that can be dealt with experimentally, for example, by including a small rotation of the beam or sample stage, or through sample preparation by using ultramicrotomy. However, as long as the data are somewhat randomly sampled around the preferred orientation, this does not affect the quality of the merged data set (Smeets & Wan, 2017 ▸). Only the completeness of the data will be lower. Electron diffraction data collected on single crystals, particularly those with low symmetries, often have a missing wedge. If measures are taken by using a high-tilt-range sample holder, this is rarely detrimental for structure determination. The mordenite and GeSi-**BEC** examples show that the problem with low completeness of the data can be overcome partly by using a program like *FOCUS*.

The examples above showed that data suitable for structure determination can be extracted in the presence of unfavourable conditions. In the case of the high-symmetry cubic phases of zeolites A and Y, the data are of high enough quality for direct methods. The accuracy of the structures determined from SerialED data was established by comparison with reference structures refined against single-crystal X-ray diffraction (Grämlich & Meier, 1971 ▸) and powder neutron diffraction data (Hriljac *et al.*, 1993 ▸). The maximum deviation of the framework atoms was found to be 0.2 Å, indicating that it is in principle possible to obtain reasonably accurate structure solutions from data collected on hundreds of individual crystals, and that the merging and histogram-matching routines are effective for dealing with these data. In the cases where direct methods fail, because of low completeness (mordenite/GeSi-**BEC**) or difficult data for indexing (ECR-18), the dual-space algorithm in *FOCUS* prevailed. These data show that 100–200 good quality diffraction patterns are enough for structure determination for materials that crystallize in a high-symmetry space group. Although models obtained from SerialED data can be further refined using XRPD data to obtain more accurate crystal structures, more development is needed to make SerialED data suitable for structure refinement. In particular, the initial structure model can serve as an added constraint during the data processing. For the orientation-finding step, for example, a method based on template matching as described by Rauch and co-workers (Rauch & Dupuy, 2005 ▸; Rauch *et al.*, 2010 ▸) can be used. This may make it possible to obtain more accurate and reliable orientations, which, in turn, will enable better algorithms for integration of reflection intensities. The structure model can also be used as a target for scaling and merging the reflection intensities.

## 6. Conclusion   

We have shown that serial electron crystallography has potential as a tool for structure determination, screening and phase identification of polycrystalline materials. Our data collection procedure is fully automated and can be used to collect electron diffraction data on about 3500 crystals per hour thanks to a fast camera. The recently developed SerialMerge algorithm was found to be an effective way to merge electron diffraction data from randomly oriented crystals, and 100–200 good diffraction patterns proved to be enough for structure determination for the crystals we tested. Finally, we showed that it is in principle possible to identify a minor impurity from SerialED data because of the possibility of probing a large number of crystals. Serial electron crystallography has the potential to develop into a stand-alone technique that can be generally applicable for phase analysis and structure determination of polycrystalline materials, and samples that are prone to radiation damage, such as organic, phamaceutical and protein crystals.

The data collection software is available from http://github.com/stefsmeets/instamatic and data processing and orientation-finding code from http://github.com/stefsmeets/problematic. The diffraction data discussed in the paper have been deposited at http://dx.doi.org/10.5281/zenodo.1158421.

## Supplementary Material

Supporting information file. DOI: 10.1107/S1600576718009500/yr5034sup1.pdf

Serial electron diffraction data URL: http://dx.doi.org/10.5281/zenodo.1158421

## Figures and Tables

**Table 1:** Summary of the SerialED experiments

Sample	Area (µm^2^)	Images	Patterns	Time (min)	Patterns per hour
Zeolite A	300 × 300	442	1107	38	1748
Zeolite Y	400 × 400	788	2506	80	1880
GeSi-**BEC**	400 × 400	723	6520	103	3798
Mordenite	200 × 200	283	694	26	1601
ECR-18	300 × 300	463	780	41	1141
Co-CAU-36	200 × 200	309	500	30	1000

**Table 2:** Crystallographic data and structure determination details for the five zeolites

	Zeolite A	Zeolite Y	GeSi-**BEC**	Mordenite	ECR-18
Composition	Si_96_Al_96_O_384_	Si_192_O_384_	Si_32-n_Ge_n_O_64_	Si_40_Al_8_O_96_	Si_672_O_1344_
Space group	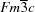	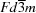	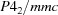	*Cmcm*	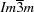
*a* (Å)	24.610	24.740	12.823	18.11	35.08
*b* (Å)	= *a*	= *a*	= *a*	20.53	= *a*
*c* (Å)	= *a*	= *a*	13.345	7.528	= *a*
*V* (Å^3^)	14905.1	15142.6	2194.3	2797.0	43169.7

Structure determination	*SHELXS*	*SHELXS*	*FOCUS*	*FOCUS*	*FOCUS*

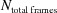	1107	2506	6520	694	780
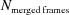	200	99	232	62	83

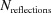	19804	11126	37467	10405	32962
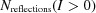	5355	7569	26144	4354	9247
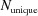	337	387	481	595	813
Completeness	100%	100%	∼70%†	∼60%†	87%
*d*_min_ (Å)	1.0	1.0	1.0	1.0	1.35

† The completeness is estimated, because exact values are highly dependent on the accuracy of the orientation finding.

**Table 3:** Deviations of the atomic positions for zeolite A and zeolite Y with respect to those obtained from structure refinement using single-crystal X-ray diffraction (Grämlich & Meier, 1971 ▸) and powder neutron diffraction (Hriljac *et al.*, 1993 ▸), respectively

		Atom		Position		Deviation (Å)
Zeolite A		Si1		96*i*		0.19
		Al1		96*i*		0.09
		O1		192*j*		0.06
		O2		96*i*		0.18
		O3		96*i*		0.13
		Na1		64*g*		0.35

Zeolite Y		Si1		192*i*		0.10
		O1		96*h*		0.11
		O2		96*g*		0.12
		O3		96*g*		0.18
		O4		96*g*		0.17
		Na1		32*e*		0.23
		Na2		32*e*		0.35

**Table 4:** Continuous rotation electron diffraction parameters and crystallographic details for cobalt oxide

Composition	CoO
Crystal system	Hexagonal
Space group	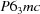
*a* (Å)	3.101 (18)
*c* (Å)	5.54 (2)
*Z*	2
λ (Å)	0.0251
Tilt range (°)	34.45 to −13.79
Frames	208
Oscillation angle (°)	0.23
Acquisition time (seconds per frame)	0.5
Completeness (%)	37.0
Observed reflections	17
Unique reflections/total	10/27
