# Supplementary material for: Serial electron crystallography for structure determination and phase analysis of nanocrystalline materials
Source: J Appl Crystallogr. 2018 Aug 9;51(Pt 5):1262–73. doi: 10.1107/S1600576718009500 (PMC6157704; doi:10.1107/S1600576718009500)
Supplement: Supplementary file 1 [file j-51-01262-sup1.pdf]

Appl Cryst JAC JOURNAL OF APPLIED CRYSTALLOGRAPHY

Volume 51 (2018)

Supporting information for article:

Serial electron crystallography for structure determination and phase analysis of nanocrystalline materials

Stef Smeets, Xiaodong Zou and Wei Wan

S1. Calibration

The data collection for a large part depends on the stability of the calibration of the position of the incident beam in imaging and primary beam in diffraction mode. In this paragraph, the calibration of the 65 experiments that have been performed over the last year in our lab are discussed. The calibration is defined by three affine matrices, M_{B1}, M_{B2}, and M_D that can be decomposed in an angle (θ) and two scale (s_x, s_y) parameters. The variation of their values are shown in Figure S4, Figure S5, and Figure S6, and summarized in Table S1.

The calibration of M_{B1} is the most critical one, because it determines whether a crystal will be hit by the beam or not, whereas the calibrations of M_{B2} and M_D merely ensure that the diffraction pattern is somewhat centered on the camera. The calibration of M_{B1} is dependent on the magnification used and is stable between experiments, resulting in low standard deviations for θ [15.668(88)°], s_x [-9.022(65)], and s_y [8.139(20)]. Some of the deviations in the angle can be attributed to the choice in camera length (400 mm instead of 250 mm). After experiment 47, a sharp drop in the value of s_y is observed that can be correlated to the installation and subsequent tweaking of a new filament.

For the calibration of M_{B2}, we observe a large degree of variation between experiments, because it depends on a large number of factors, such as the actual alignment of the beam, the camera length, and the size of the beam. This is reflected in the large standard deviations on the values of θ [16.7(1.0)°], s_x [-4.6(1.4)], and s_y [4.4(1.3)]. This indicates that the calibration for M_{B2} should be repeated before every experiment.

The calibration of M_D appears to be the most stable one, and barely deviates over the 65 experiments. This is reflected in the very low standard deviations for θ [4.950(97)°], s_x [-3.5616(67)], and s_y [4.0958(59)]. Some of the deviations in the angle can be attributed to the choice in camera length (400 mm instead of 250 mm). As with M_{B1}, deviations in the angle can be attributed to the choice in camera length, and in the scale to the installation and tweaking of a new filament.

S2. Structure determination of GeSi-BEC

GeSi-**BEC** is a large-pore germanosilicate zeolite with a tetragonal ($P4_2/mmc$) unit cell [Corma, A., Navarro, M. T., Rey, F., Rius, J. & Valencia, S. (2001). Angew. Chem. 113, 2337–2340] that was first synthesized as a pure germinate [Conradsson, T., M. S. Dadachov, & X. Zou. (2000). *Micropor. Mesopor. Mat.* 41, 183–91]. For one of the samples, a data set consisting of 6520 diffraction patterns were collected over an area of 400 × 400 μm in 103 minutes. The crystals were ideally distributed on the grid; isolated, but with a high density. These data show that in case of a dense sample distribution, data collection rates of nearly 4000 diffraction patterns per hour are attainable. As was the case with

the mordenite sample, Ge-BEC also has a problem with preferred orientation. After the orientation finding and indexing procedure, the 232 best frames were selected for structure determination using the structure of ZSM-5 (**MFI**) as a source of intensities for the reflections after merging. As a result of the lowered completeness, structure determination using *ShelXS/ShelXT* failed. However, as with the mordenite sample, the structure could be solved in a straightforward manner using the program FOCUS.

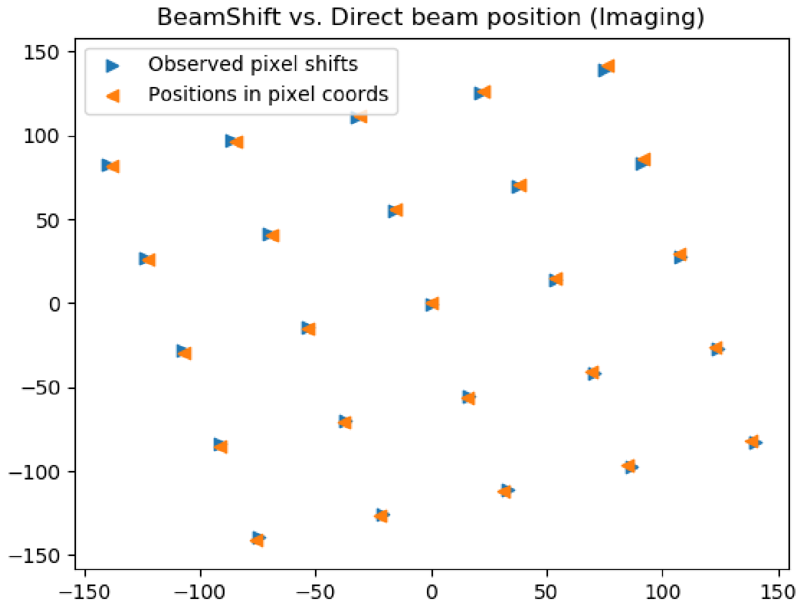

Figure S1 Typical calibration of the beam shift in imaging mode.

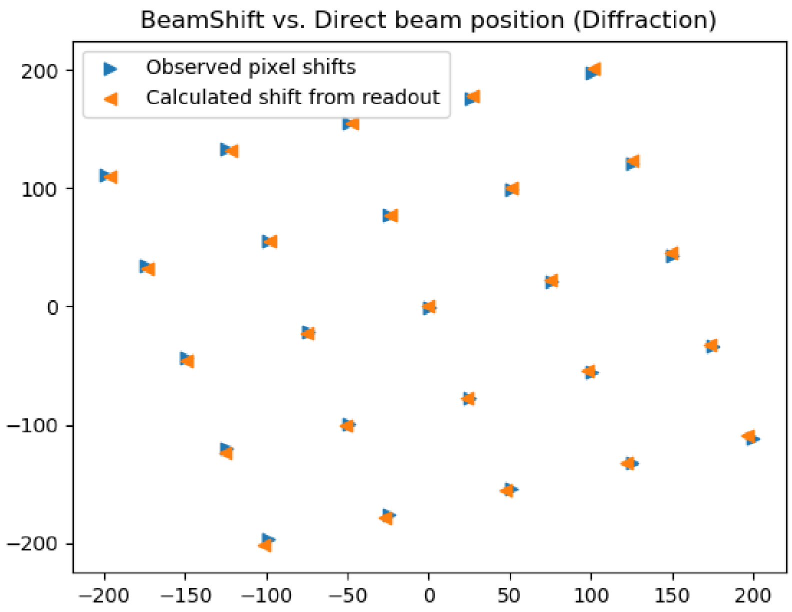

Figure S2 Typical calibration of the beam shift in diffraction mode.

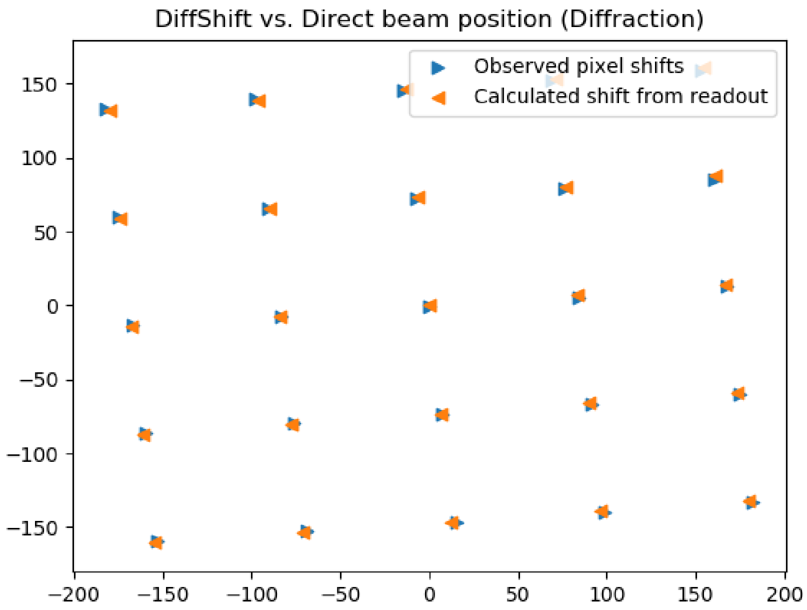

Figure S3 Typical calibration of the diffraction shift in diffraction mode.

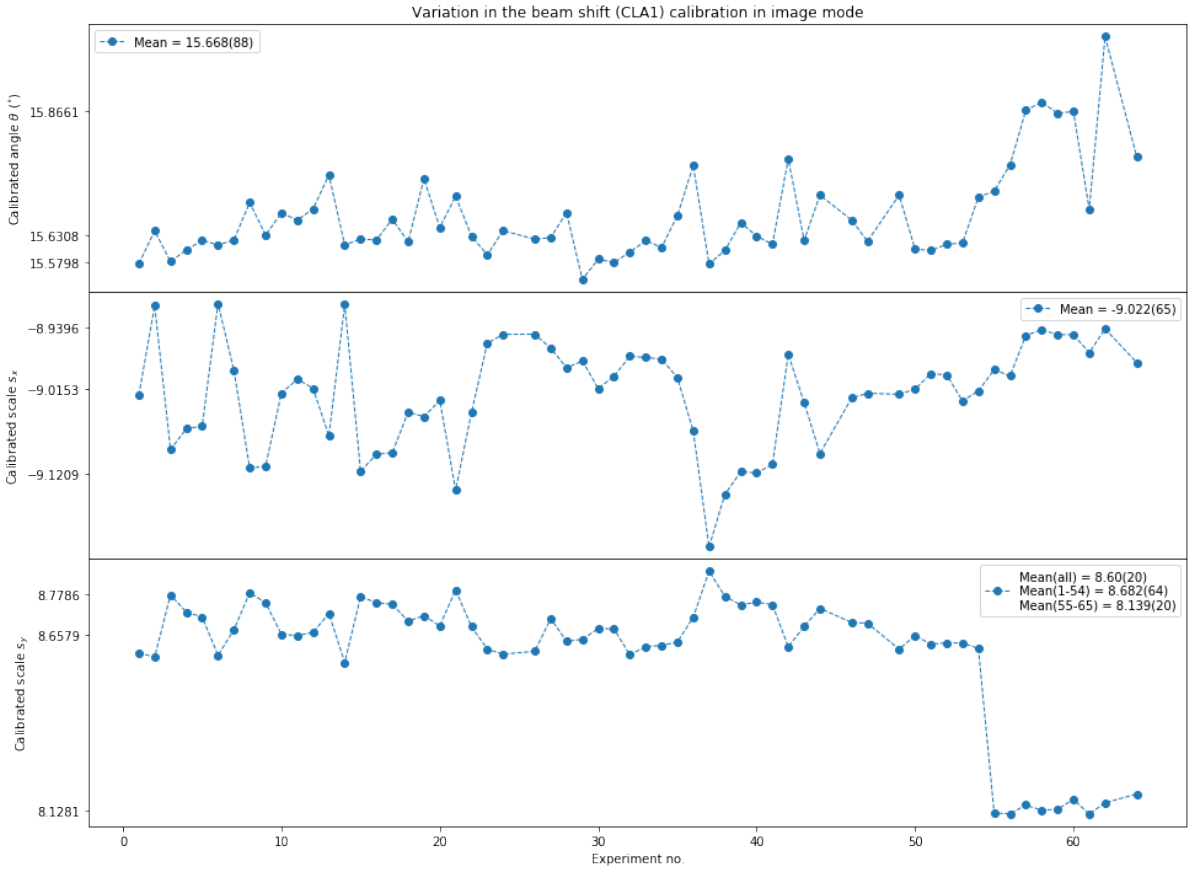

Figure S4 Variation of the angle (θ) and scale (s_x, s_y) relating the position of the probe (incident beam) in *image mode* (M_{B1}; magnification: 2500×) in pixel coordinates to the values of the beam shift deflectors (CLA1) for 65 experiments taken over the course of a year. Four outliers (experiment 24, 44, 47, 62) have been omitted from this plot, two of these can be attributed to a different choice in magnification (frame 44: 3000×, frame 47: 4000×), which has a significant effect on the scale parameters. The legend shows the mean calibrated values and their standard deviations. For the s_y scale, mean values have been calculated independently for the first 53 and last 11 frames, because the calibration appears to have changed starting experiment 55 after tweaks to the settings of a new filament that was installed between experiment 47 and 48. The tick marks on the *y*-axis correspond to the median, 5th and 95th percentile values.

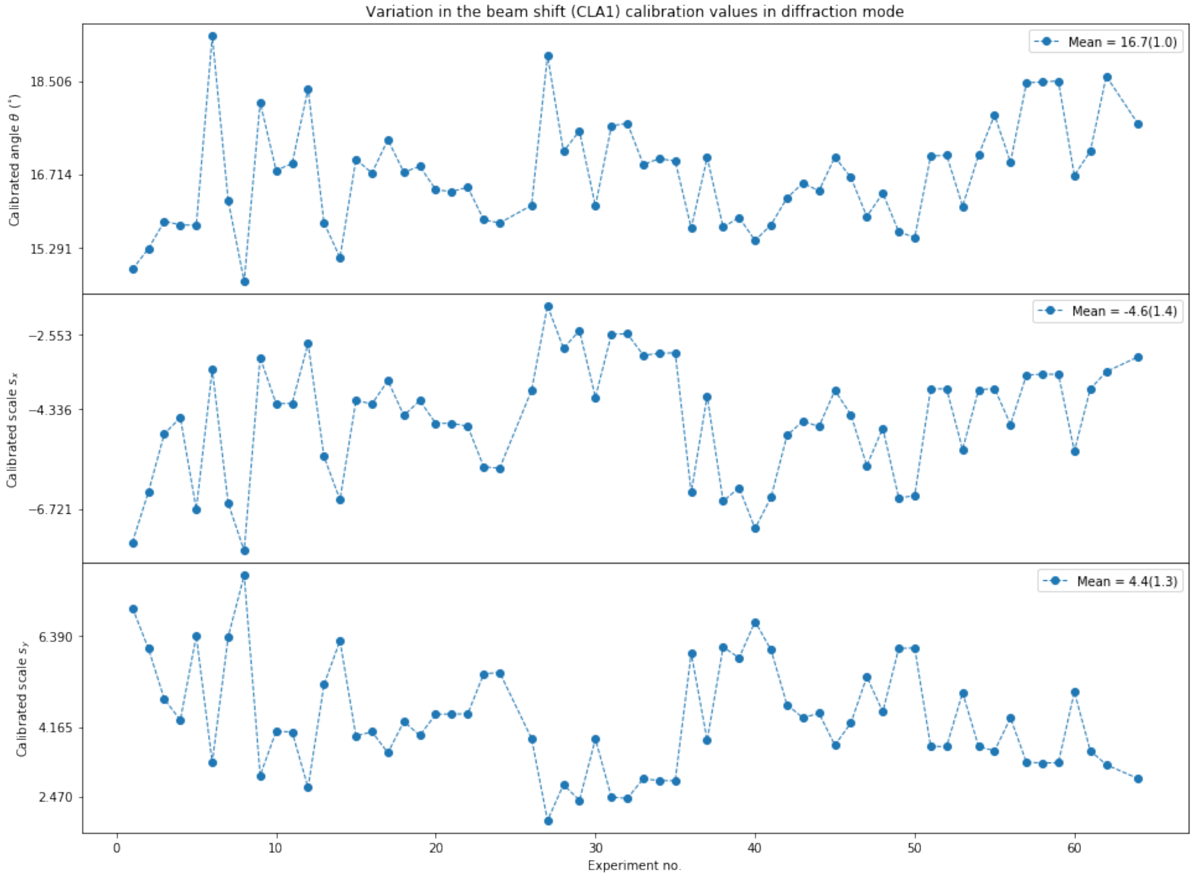

Figure S5 Variation of the angle (θ) and scale (s_x, s_y) relating the position of the probe (incident beam) in *diffraction mode* (M_{b2}) in pixel coordinates to the values of the beam shift deflectors (CLA1) for 65 experiments taken over the course of a year. Two outliers (experiment 24, 62) have been omitted from this plot. The legend shows the mean calibrated values and their standard deviations. The tick marks on the *y*-axis correspond to the median, 5th and 95th percentile values.

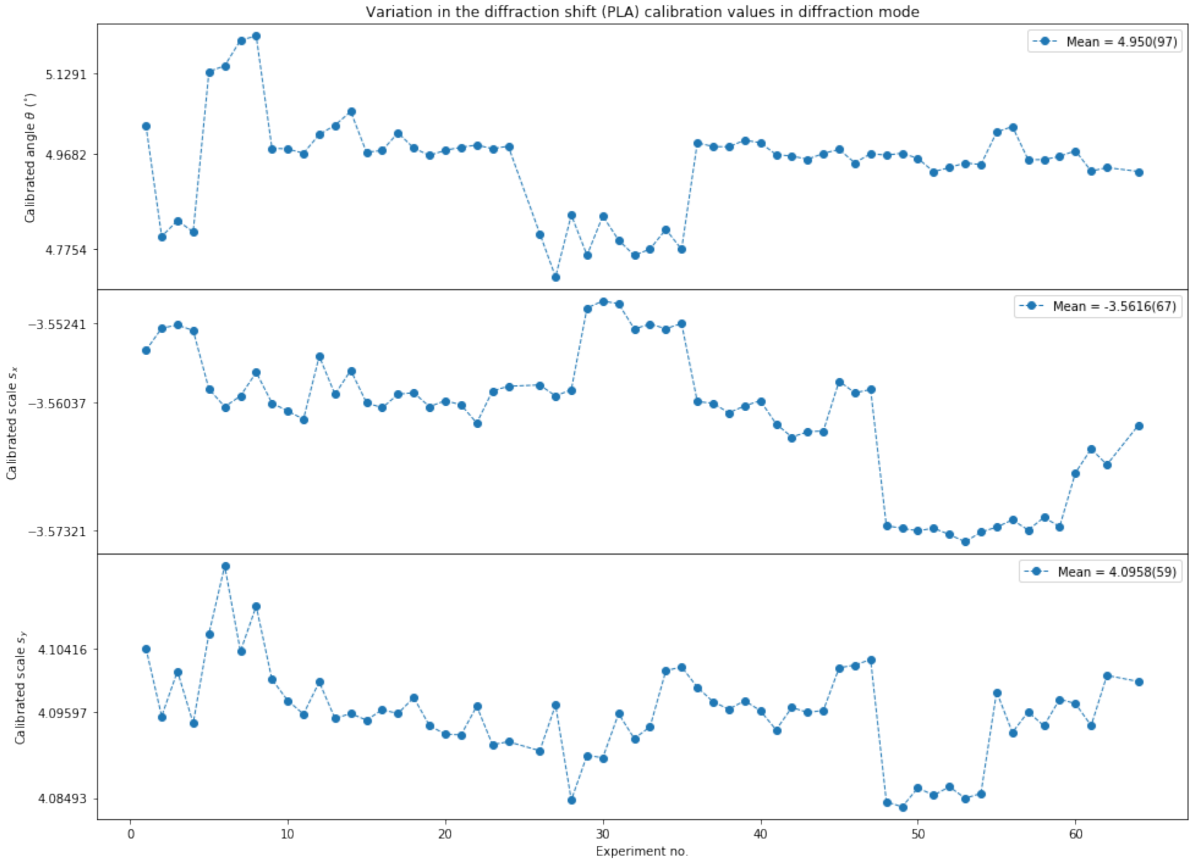

Figure S6 Variation of the angle (θ) and scale (s_x, s_y) relating the position of the probe (incident beam) in *diffraction mode* (M_D; camera lengths: 200-400 mm) in pixel coordinates to the values of the diffraction shift deflectors (PLA) for 65 experiments taken over the course of a year. Two outliers (experiment 24, 62) have been omitted from this plot. The legend shows the mean calibrated values and their standard deviations. The tick marks on the *y*-axis correspond to the median, 5th and 95th percentile values. Some of the deviations in the angle can be attributed to the choice in camera length (frames 2-4, 25-34 were taken at 400 mm), although the difference is negligible. The drops in s_x and s_y observed between experiment 47 and 48 can be correlated to the installation of a new filament, and the increase in s_y in experiment 55 to tweaks to the settings of the filament.

Table S1 Mean calibrated values for the angle (θ) and scale (s_x, s_y) parameters relating the lens values to observed position of the primary beam.

	Experiment	θ (°)	s_x	s_y
CLA1 (image, M_{B1})	All	15.668(88)	-9.022(65)	8.60(20)
	1-54	-	-	8.682(64)
	55-64	-	-	8.139(20)
CLA1 (diff., M_{B2})	All	16.7(1.0)	-4.6(1.4)	4.4(1.3)
PLA (diff., M_D)	All	4.950(97)	-3.5616(67)	4.0958(59)
